# SigE: A master regulator of *Mycobacterium tuberculosis*

**DOI:** 10.3389/fmicb.2023.1075143

**Published:** 2023-03-07

**Authors:** Riccardo Manganelli, Laura Cioetto-Mazzabò, Greta Segafreddo, Francesca Boldrin, Davide Sorze, Marta Conflitti, Agnese Serafini, Roberta Provvedi

**Affiliations:** ^1^Department of Molecular Medicine, University of Padova, Padova, Italy; ^2^Department of Biology, University of Padova, Padova, Italy

**Keywords:** *Mycobacterium tuberculosis*, sigma factor, stress response, regulatory network, pathogenesis

## Abstract

The Extracellular function (ECF) sigma factor SigE is one of the best characterized out of the 13 sigma factors encoded in the *Mycobacterium tuberculosis* chromosome. SigE is required for blocking phagosome maturation and full virulence in both mice and guinea pigs. Moreover, it is involved in the response to several environmental stresses as surface stress, oxidative stress, acidic pH, and phosphate starvation. Underscoring its importance in *M. tuberculosis* physiology, SigE is subjected to a very complex regulatory system: depending on the environmental conditions, its expression is regulated by three different sigma factors (SigA, SigE, and SigH) and a two-component system (MprAB). SigE is also regulated at the post-translational level by an anti-sigma factor (RseA) which is regulated by the intracellular redox potential and by proteolysis following phosphorylation from PknB upon surface stress. The set of genes under its direct control includes other regulators, as SigB, ClgR, and MprAB, and genes involved in surface remodeling and stabilization. Recently SigE has been shown to interact with PhoP to activate a subset of genes in conditions of acidic pH. The complex structure of its regulatory network has been suggested to result in a bistable switch leading to the development of heterogeneous bacterial populations. This hypothesis has been recently reinforced by the finding of its involvement in the development of persister cells able to survive to the killing activity of several drugs.

## Introduction

*Mycobacterium tuberculosis*, the etiologic agent of tuberculosis, is a formidable obligate pathogen who coevolved with human beings for millennia (McHenry et al., 2020). Its genome is extremely stable (Gagneux, 2018), probably due to its slow growth rate, and can be considered as the result of this long process of coevolution and adaptation to its natural environment: our body. Tuberculosis is a very complex disease, during which bacteria are exposed to several different intracellular and extracellular environments, each characterized by specific nutritional and stressing peculiarities to which bacteria must necessarily adapt.

Adaptation of bacteria to changing environments is mostly regulated at the transcriptional level through transcriptional regulators able to sense and react to environmental cues. One of the most represented class of bacterial transcriptional regulators is that of sigma factors. These are proteins able to associate to the RNA polymerase (RNAP) core enzyme to confer promoter specificity. All bacterial genomes encode at least one primary sigma factor, which is essential to allow the RNA polymerase holoenzyme to recognize the promoters of housekeeping genes. However, usually bacterial genomes encode several alternative sigma factors, which are transcriptionally or post-translationally activated in response to specific stimuli. Upon activation, alternative sigma factors can compete with the primary sigma factor to form an alternative RNA polymerase holoenzyme able to recognize a different set of promoters allowing the bacterial cell to shift very quickly among different transcriptional programs (Abril et al., 2020). Usually, environmental bacteria, or bacteria with peculiar developmental program (e.g., sporulation) have a higher alternative sigma factor chromosomal density (number of alternative sigma factors/Mb) than pathogenic bacteria (Rodrigue et al., 2006).

The *M. tuberculosis* chromosome encodes 13 sigma factors and is the bacterial obligate pathogen with the highest alternative sigma factors chromosomal density (Rodrigue et al., 2006; Manganelli, 2014), confirming the peculiarity and the complexity of its relationship with its host.

One of the best characterized alternative sigma factors of *M. tuberculosis* is SigE, belonging to the extracellular function (ECF) sigma factor family.

## Role of SigE in *Mycobacterium tuberculosis* physiology and virulence

Transcription of *sigE* is induced upon exposure to several different conditions as heat shock, acidic or alkaline pH, oxidative stress, detergents, vancomycin, hypoxia, phosphate starvation and during infection of human macrophages (Manganelli et al., 2001; Schnappinger et al., 2003; He et al., 2006; Sureka et al., 2007; Provvedi et al., 2009; Kundu and Basu, 2021). Accordingly, a *M. tuberculosis sigE* null mutant was more sensitive to different stress conditions, such as surface stress, heat shock, oxidative stress and to treatment with several antibacterial drugs (Manganelli et al., 2001). It was also unable to grow in human dendritic cells (Giacomini et al., 2006) and in THP-1-derived human macrophages (Manganelli et al., 2001), while it was still able to grow in the human pneumocyte cell line A549 (Casonato et al., 2014). Moreover, it was unable to block phagosome maturation in human macrophages (Casonato et al., 2014) and more sensitive to the bactericidal activity of activated murine macrophages (Manganelli et al., 2001). Finally, it was strongly attenuated in both mice (Manganelli et al., 2004) and guinea pigs (Troudt et al., 2017).

DNA microarrays were used to determine the SigE regulon following surface stress and during macrophage infection (Manganelli et al., 2001; Fontán et al., 2008). Briefly, genes under the control of SigE were shown to be related to metabolic remodeling associated to stress-mediated growth arrest, fatty acid degradation, membrane proteins quality-control and membrane stabilization (Manganelli et al., 2001; Manganelli, 2014), suggesting that the surface structure of the *sigE* mutant might have different characteristics and composition than that of the wt strain. The different surface structure and the inability to block phagosome maturation suggest that the mutant strain has different immunogenic properties compared to the wt. This was confirmed by several evidences as the induction of IL-10 production in dendritic cells (Giacomini et al., 2006), or the different transcriptional profile of macrophages infected with the *sigE* mutant compared to that of macrophages infected with *M. tuberculosis* wt (Fontán et al., 2008).

Despite the very low bacterial burden, mice infected with a *sigE* null mutant showed higher production of protective factors as gamma interferon, tumor necrosis factor alpha, inducible nitric oxide synthase, and beta-defensins, compared to mice infected with wt *M. tuberculosis* suggesting the possibility to use this mutant as an attenuated vaccine (Pando et al., 2010). Indeed, both mice and guinea pigs vaccinated subcutaneously with a *sigE* mutant showed protection from infection with wt *M. tuberculosis* equal or even higher than animals vaccinated with BCG (Pando et al., 2010; Troudt et al., 2017). To fulfill the criteria of the Geneva Consensus for entering human clinical trials, we recently constructed a *fadD26-sigE* double mutant which was shown to be more attenuated and more efficacious of BCG in different mouse models of infection and similar to BCG in a guinea pig model of infection (Hernandez-Pando et al., 2019).

## The *sigE* locus

**Figure 1:**
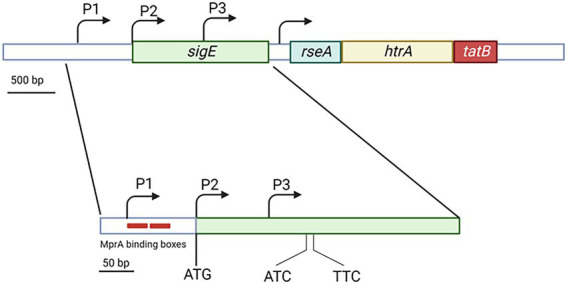
Structure of the *sigE* genomic region showing the *sigE* coding sequence with its 3 promoters and the *rseA-htrA-tatB* operon. While P2 coincides with the main *sigE* translational start codon, P3 is internal to *sigE* coding region. Trancripts from this promoters are translated from the two indicated uncanonical promoters. The two red boxes in correspondence of P1 represent the MprA binding boxes. Created with BioRender.com.

The *sigE* gene is followed by *rseA* encoding for the SigE-specific anti sigma factor (Boldrin et al., 2019), a protein able to bind SigE in the absence of stress preventing its interaction with the RNA polymerase holoenzyme. Usually, sigma factor and anti sigma factor genes are encoded in operons and though cotranscribed. However *sigE* and *rseA*, even if adjacent, are not cotranscribed, and a promoter is present in their intergenic region (Figure 1). This can have important implications in the post-translational regulation of SigE, since the differential regulation of the two genes can result in modification of the ratio between the two proteins and consequently a different amount of free SigE. Interestingly, *rseA* lays in the same operon with the two next genes: *htrA* and *tatB*. The first encodes an essential serine protease involved in the degradation of a cell wall amidase (Wu et al., 2019), while the second encodes an integral membrane protein belonging to the two-arginine translocation system. The physiological meaning of the coexistence of these three genes in the same operon is still difficult to understand (Manganelli, 2014).

The gene encoding SigE is transcribed from three different promoters: P1, P2, and P3. P1 is active in normal physiologic conditions and represents the *sigE* housekeeping promoter, probably recognized by a σ^A^-RNA polymerase. P2 originates a leaderless mRNA, its activation requires the two-component system MprAB, and is transcribed by SigE-RNA polymerase. Finally, P3, which is located inside the *sigE* coding region, is recognized by σ^H^-RNA polymerase. The mRNA resulting from P3 is translated from two noncanonical translational start codons within the *sigE* coding sequence resulting in the translation of two smaller SigE isoforms (Figure 1; Donà et al., 2008; Chauhan et al., 2016).

## Surface stress

When *M. tuberculosis* is exposed to surface stress, *sigE* is strongly induced. Accordingly, a *sigE* null mutant is more sensitive to detergents than its parental strain (Manganelli et al., 2001). The mechanism of *sigE* induction and SigE activation in conditions of surface stress has been characterized, and is very complex, relying on several feed forward loops (FFL), the first of which is due to SigE autoregulation (Chauhan et al., 2016). Two main sensors govern the SigE mediated surface stress: the two-component system MprAB and the protein kinase B (PknB). The first is responsible for *sigE* transcriptional regulation, while the second is responsible for its post-translational regulation (Manganelli and Provvedi, 2010).

**Figure 2:**
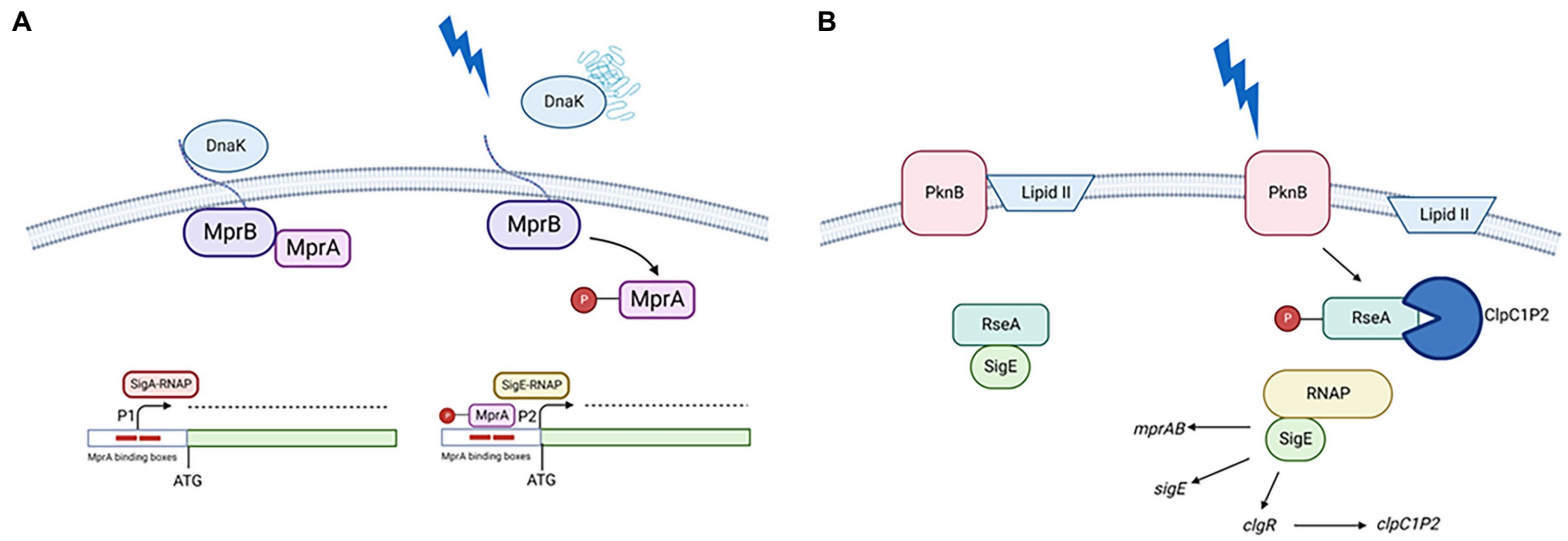
**(A)** Transcriptional activation of P2. In the presence of unfolded proteins in the periplasm, DnaK releases the extracellular domain of MprB leading to MprA phosphorylation. Phosphorylated MprA binds to its operators turning off P1 and turning on P2; **(B)** Post translational activation of SigE. In the presence of surface stress PknB phosphorylates RseA, probably due to a transient interference of the interaction between its PASTA domain and Lipid II. Once phosphorylated, RseA is targeted for degradation by the ClpC1P2 protease releasing the active form of SigE. This will favor the induction its regulon which includes *clgR*, encoding a positive regulator of the *clp* genes. Created with BioRender.com.

In physiologic conditions the extracytoplasmic domain of the sensor kinase MprB is bound to the chaperone DnaK, which downregulates its activity. When due to surface stress, unfolded or misfolded proteins appear in the extracytoplasmic space, they outcompete the MprB extracytoplasmic domain in DnaK binding, leading to the activation of its kinase activity and MprA phosphorylation (Bretl et al., 2014). Phosphorylated MprA can bind its binding boxes in the *sigE* promoter regions (He et al., 2006) turning off P1 and on P2 (Donà et al., 2008). Since *mprAB* promoter is under SigE transcriptional control, its expression will increase, thus increasing the amount of MprAB and consequently that of P2 activation (second FFL; Manganelli et al., 2001; Figure 2A).

PknB (the second sensor) in condition of surface stress phosphorylates a tyrosine residue of the anti-sigma factor RseA, which consequently can be recognized and degraded from the ClpC1P2 complex. Since the expression of genes encoding the ClpC1P2 complex are positively regulated by ClgR, a pleiotropic regulator which is part of the SigE regulon, this represent a third FFL (Barik et al., 2010; Manganelli and Provvedi, 2010; Figure 2B). The mechanism activating RseA phosphorylation by PknB following stress response is still not elucidated. PknB is an essential gene able to modulate the activity of several proteins involved in essential pathways as cell-wall synthesis, cell growth and metabolism. Recently, it was demonstrated that the interaction of its PASTA domain with Lipid II is essential for its correct localization at the cell poles and for its correct activation guarantying cellular homeostasis (Kaur et al., 2019). Interestingly, it was shown that preventing the interaction of Lipid II with the PASTA domains led not only to an incorrect localization of the protein, but also to its hyperactivation with consequent phosphorylation of proteins not usually considered PknG substrates, leading to loss of cellular homeostasis and eventually cell death (Kaur et al., 2019). It is possible to hypothesize that surface stress could transiently interfere with the interaction between Lipid II and PknG PASTA domain leading to RseA phosphorylation.

Mathematical modeling of this complex regulatory network underlined the importance of regulation of MprAB activity by DnaK, and of fine regulation of RseA concentration in the outcome of the system, suggesting that the interaction among the several positive feedback loops part of the network, could result in bistability (Tiwari et al., 2010; Rao et al., 2021; Zorzan et al., 2021).

We recently challenged this model studying the dynamics of induction of the system to determine the role and hierarchy of the two pathways. At this purpose, we compared the expression of *sigE* and of two genes under SigE transcriptional control (*sigB*, and *clgR*) in different mutants and found that both the MprAB and the PknB pathways were equally essential for the activation of surface stress response in *M. tuberculosis*. Interestingly, when the same experiments were performed in *Mycobacterium smegmatis* the results were different. In this species, the MprAB pathway was clearly dominant, while the PknB pathway was secondary (Cioetto-Mazzabò, et al. submitted).^^1^^

## Oxidative stress

**Figure 3:**
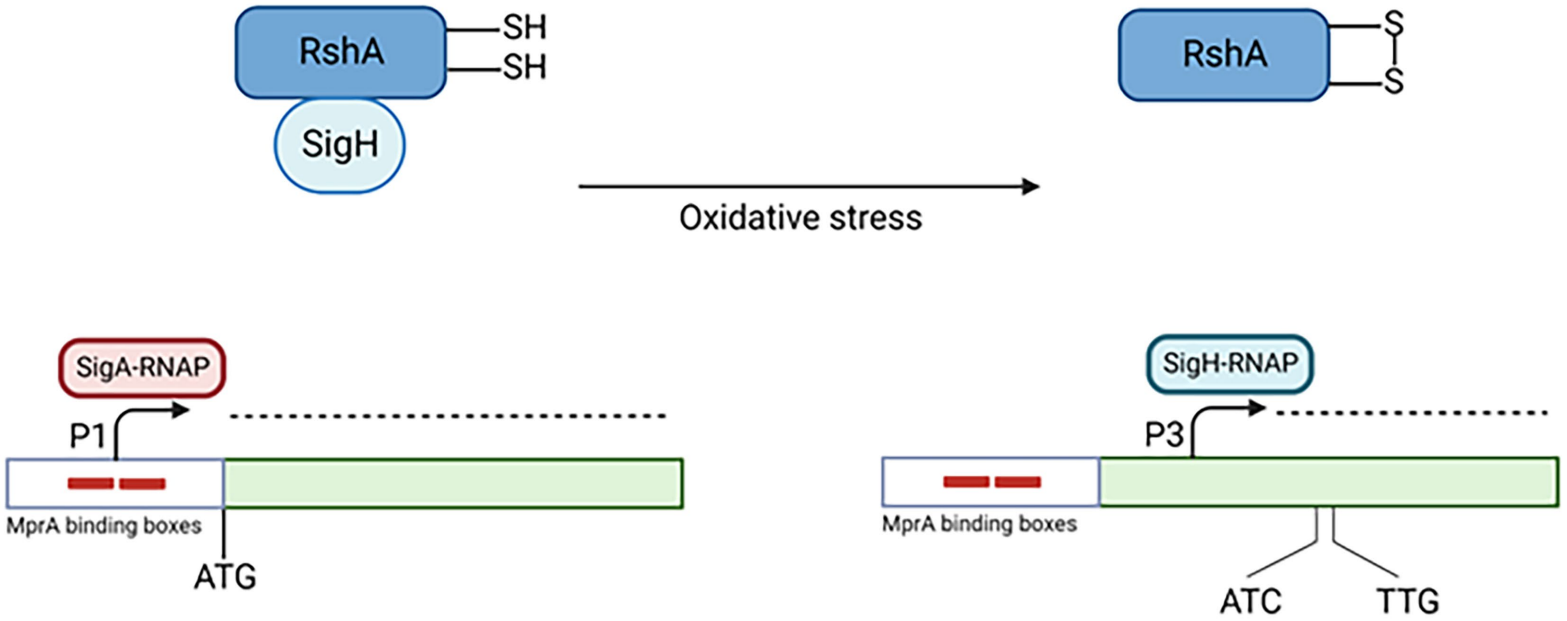
Transcriptional activation of P3. In conditions of oxydative stress, σ^H^ is released by its antisigma factor RshA. Consequently, it binds the RNAP core enzyme and transcribes the genes belonging to its regulon, including *sigE* P3. Created with BioRender.com.

*sigE* is also induced in oxidative environment, where a *sigE* null mutant survives less than its parental wt strain (Manganelli et al., 2002). The mechanism of induction in this condition is simpler than that functioning following surface stress, and relies principally on another ECF sigma factor: σ^H^. This sigma factor is regulated by the antisigma factor RshA, which releases σ^H^ in oxidative environment (Song et al., 2003). σ^H^-RNA polymerase is able to initiate transcription at P3, a transcriptional start site inside the SigE coding region, producing a shorter mRNA that can be translated from two alternative translational start sites. Consequently, in condition of oxidative stress two shorter isoforms of SigE are produced (215 and 218 aa *vs* 257 aa of the major isoform). Preliminary studies were not able to reveal main differences in the functionality of these SigE isoforms and the main isoform of SigE (Donà et al., 2008; Figure 3).

## Low pH

**Figure 4:**
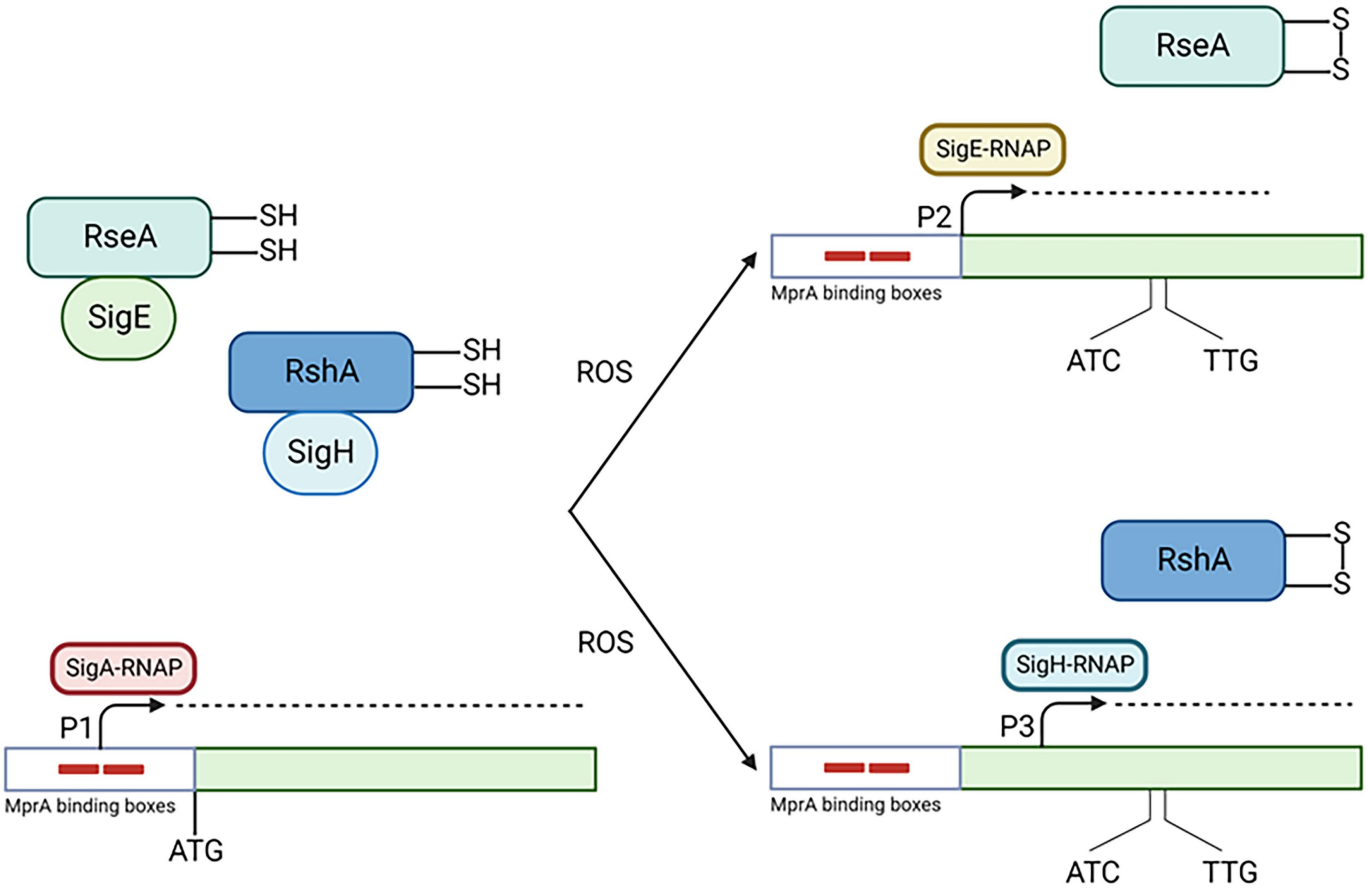
Activation of the SigE network in condition of low pH. Low pH induces damages in periplasmic proteins leading to MprA phosphorylation (not shown). Production of ROS due to low pH oxidize RseA, which consequently release SigE which associate with RNAP core enzyme and transcribe its own gene from P2 with the help of MprA. This helps the cell to limit the production of ROS. At later time points or in the absence of SigE activation (as in the MprAB mutant), ROS accumulates leading to the release of σ^H^ from its anti sigma factor RshA and consequently to the activation of *sigE* P3. Created with BioRender.com.

SigE is part of *M. tuberculosis* response to low pH, which is very complex, involves several regulators and is still not fully characterized (Baker et al., 2019). We recently characterized the *sigE* pathway of induction during the first 90 min of exposure to low pH, determining that MprAB-dependant activation of P2 plays a fundamental role in its activation from very early time points (15 min post-exposure; Cioetto-Mazzabò et al., submitted). It is possible that low pH can determine the unfolding of periplasmic proteins leading to the release of DnaK from MprB and consequently its activation. However, in a mutant missing this two component system, *sigE* is still induced, but its transcription starts from the σ^H^-dependant promoter P3, and is delayed of 15 min. At low pH, bacteria experience reductive stress, and this has been demonstrated to cause the formation of reactive oxygen species (ROS), paradoxically resulting in enhanced oxidative stress and activation of σ^H^ after 4 h of exposure (Coulson et al., 2017; Baker et al., 2019). It is possible that immediately after exposure to low pH, the induction of the SigE regulon can counteract the effects of reductive stress, limiting the production of ROS, which accumulates at later time points leading to the induction of *sigH*. However, in the absence of MprAB, the SigE network is not activated and ROS are accumulated early after exposure, leading to an earlier activation of σ^H^ (Figure 4).

Regarding post-translational regulation, degradation of RseA plays a minor role in releasing active SigE, since in a mutant where RseA cannot be phosphorylated by PknB, or in a mutant missing ClgR the activation of the SigE network is only mildly attenuated, though still present and dependent on P2. *clgR* is cotranscribed with other three genes encoding the Phage-Shock Protein (Psp) system; ClgR is bound and kept in an inactive form by PspA, until following surface stress, PspA releases it to bind the surface-associated proteins Rv2743c and Rv2742c through a partner-switching mechanism (Manganelli and Gennaro, 2017). It is possible that in condition of low pH this mechanism is only mildly activated, with ClgR remaining inactive and unable to induce the transcription of *clp* genes. This suggests that the main pathway of SigE post translational regulation in conditions of low pH is based on the ROS sensitivity of RseA (Barik et al., 2010): we can hypothesize that in the wt strain the production of ROS caused by low pH, even if not reaching the level reached in the *mprAB* mutant and needed to activate σ^H^, is high enough to oxidize RseA, abrogating its interaction with SigE.

## Low phosphate and stringent response

Stringent response plays a fundamental role in reshaping host metabolism in condition of starvation, allowing bacteria to slow down replication to save energy and survive until nutrients are newly available. The development of stringent response is due to the alarmone (p)ppGpp, produced by the protein Rel after sensing intracellular signals such as stalling ribosomes (Irving and Corrigan, 2018). Usually bacteria in this situation are more tolerant to antibiotics and different stressing conditions (Pacios et al., 2020). The most characterized experimental conditions used to reproduce stringent response in *M. tuberculosis* is growth on low phosphate (Rifat et al., 2009). In these conditions *M. tuberculosis* stops its growth, become more tolerant to drugs (Dutta et al., 2019; Danchik et al., 2021), and increases capsular polysaccharides production (van de Weerd et al., 2016). A *sigE* null mutant was able to survive in these conditions, but was unable to increment capsular polysaccharide production (van de Weerd et al., 2016). Since a likely SigE consensus sequence is present upstream the *rel* gene, it had been proposed that *sigE* induction in presence of low phosphate results in *rel* induction and activation of the stringent response. In a recent work designed to challenge this hypothesis, we analyzed by RNA-seq the transcriptional response to low phosphate of a *sigE* mutant and its parental strain at different time points. Interestingly, we could observe the typical features of stringent response in both strains, suggesting that SigE is not the key factor to activate this kind of response. However, we found that indeed low phosphate concentration led to the activation of the SigE regulon, which was not activated in the *sigE* mutant, where it was replaced by the strong induction of the oxidative stress-specific sigma factor σ^H^ (Baruzzo et al., 2023). These data suggest that the main role of SigE in low phosphate is not that to activate the stringent response inducing *rel*, but that to protect the bacterial cells from the stress caused by the decrease and remodeling of bacterial metabolism caused by stringent response. The discrepancy between these data and those suggesting a direct involvement of SigE in *rel* activation can be explained by the fact that the latter were mainly obtained working on *M. smegmatis* and not in *M. tuberculosis* (Sureka et al., 2007). Unfortunately, no information on the mechanism of activation of the network are available in these conditions.

RNA-seq data showing the induction in both the *sigE* mutant and in its parental wt strain of other genes related to surface stress, but not dependent on SigE, suggests that the fast metabolic shutdown experienced in these conditions by the bacteria might lead to membrane damages activating SigE (Baruzzo et al., 2023).

## Hypoxia and persistence

One of the main features of *M. tuberculosis* is its ability to develop a dormant state in response to the conditions found during infection that allow the bacteria to survive for decades in the human body to eventually reactivate when the pressure of the immune response decreases. Despite an enormous amount of studies, the lack of a reliable animal model prevented the possibility to establish the mechanism used by *M. tuberculosis* to accomplish this goal. However, several *in vitro* models using conditions encountered by the bacteria during infection and causing *M. tuberculosis* to develop a dormancy-like phenotype have been developed. Among them the hypoxia model is the best characterized. In this model bacteria are grown in sealed bottles in which the amount of oxygen slowly decreases due to its consumption by the bacteria (Wayne and Hayes, 1996). In response to oxygen depletion, *M. tuberculosis* enacts a complex transcriptional response leading to profound changes in its metabolism mainly led by the products of the DosR regulon (Du et al., 2016; Kundu and Basu, 2021). Dormant bacteria obtained with this method can survive for long periods of time and become tolerant to several drugs. *sigE*, as well as *sigB* and *sigH*, have been shown to be induced during oxygen depletion (Veatch and Kaushal, 2018; Kundu and Basu, 2021), even if different groups reported this induction at different time points, probably for the difficulty to obtain exactly the same experimental conditions. Dormant bacteria, when re-exposed to oxygen can reactivate starting to divide again. During this phase, same authors reported induction while others reported repression of *sigE* (Du et al., 2016; Veatch and Kaushal, 2018). Also in this case these discrepancies were probably due to different experimental conditions. However, the importance of SigE in this phenomenon has been clearly shown, since a *sigE* null mutant was shown to reactivate slower than the wt strain (Du et al., 2016). The mechanism used by *M. tuberculosis* to induce *sigE* in hypoxia condition has not been investigated yet. However, the activation in these condition of several genes of the MprAB and σ^H^ regulons (Du et al., 2016), suggests that in these conditions *sigE* might be activated by both pathways. After the transition period, when the oxygen concentration is stable and bacterial replication completely stops, probably the expression of *sigE* will return at the basal level. During reactivation, when oxygen is again available, cells would probably experience an increasing oxidative stress and this would activate again the σ^H^ pathway that would cause *sigE* induction.

Other models to study dormancy have been developed, based on starvation (Betts et al., 2002), vitamin C (Nandi et al., 2019) or potassium depletion (Salina et al., 2019) and *sigE* transcription was shown to be induced in all of them, underlining the importance of this sigma factor in governing the mechanisms leading to the transition between active growth and dormancy (Boldrin et al., 2020).

## Role of SigE in tolerance and persistence to drugs

The role of stress response mechanisms and ECF sigma factors in antimicrobials tolerance, persistence and resistance is well recognized (Harms et al., 2016; Woods and McBride, 2017). In a previous work, we compared the sensitivity and the ability to persist to bactericidal drugs of a *sigE* null mutant of *M. tuberculosis* and its wt parental strains. We found that the mutant strain was more susceptible to several drugs, including isoniazide and rifampin. Moreover, the frequency of the occurrence of persisters able to escape killing from isonoazide, streptomycin and vancomycin was reduced in the mutant strain. Of interest the fact that a bacteriostatic drug as ethambutol was strongly bactericidal for the *sigE* mutant. Some of these phenotypes were also found in a *sigB* null mutant: since the expression of *sigB* is almost totally dependent on SigE, some of the phenotypes of the *sigE* mutant might be due to the down-modulation of *sigB* transcription in this strain (Pisu et al., 2017). Finally, it was recently reported that pyrazinamide susceptibility depends on the SigE-dependent activation of surface stress and that a SigE null mutant is resistant to this drug (Thiede et al., 2022).

## SigE, two-component systems and other regulators

Interaction among transcriptional regulators as two-component systems and sigma factors can increase complexity and accuracy of the transcriptional response to a changing environment. A sigma factor, once present in the cytoplasm in active form, can associate to the RNA polymerase core enzyme and start the transcription of the genes belonging to its regulon, but some of them might require a second signal in addition of the presence of the specific sigma factor to be transcribed. An example of this is the physical interaction between SigE and PhoP that was shown to be essential for SigE -mediated induction of different genes during acid stress (Bansal et al., 2017). Even if direct physical interaction between SigE and a member of the two-component systems has been demonstrated only in the case of PhoP, data suggest its interaction with at least other two two-component systems. The first is MprAB: both the *sigE* P2 promoter and the *sigB* promoter are transcribed by a SigE-RNA polymerase, but require the activation of this two-component system by surface stress for their full activation (He et al., 2006). The second is SenX3-RegX3, which is activated in condition of low phosphate and that is essential for the SigE -dependent induction of *ppk1* in these conditions (Rifat et al., 2009; Sanyal et al., 2013).

As the activity of two-component systems can modulate the activity of sigma factors including SigE, also SigE can modulate the activity of other regulators both directly and indirectly. Directly when it is responsible of the induction of their structural genes in specific conditions, as in the case of MprAB, σ^B^ or ClgR, indirectly in the case of RbpA. This protein, whose structural gene is part of the SigE regulon, can associate to σ^A^ and σ^B^ modulating their activity (Hu et al., 2016; Prusa et al., 2018; Wang et al., 2018; Vishwakarma and Brodolin, 2020).

## Conclusion

**Figure 5:**
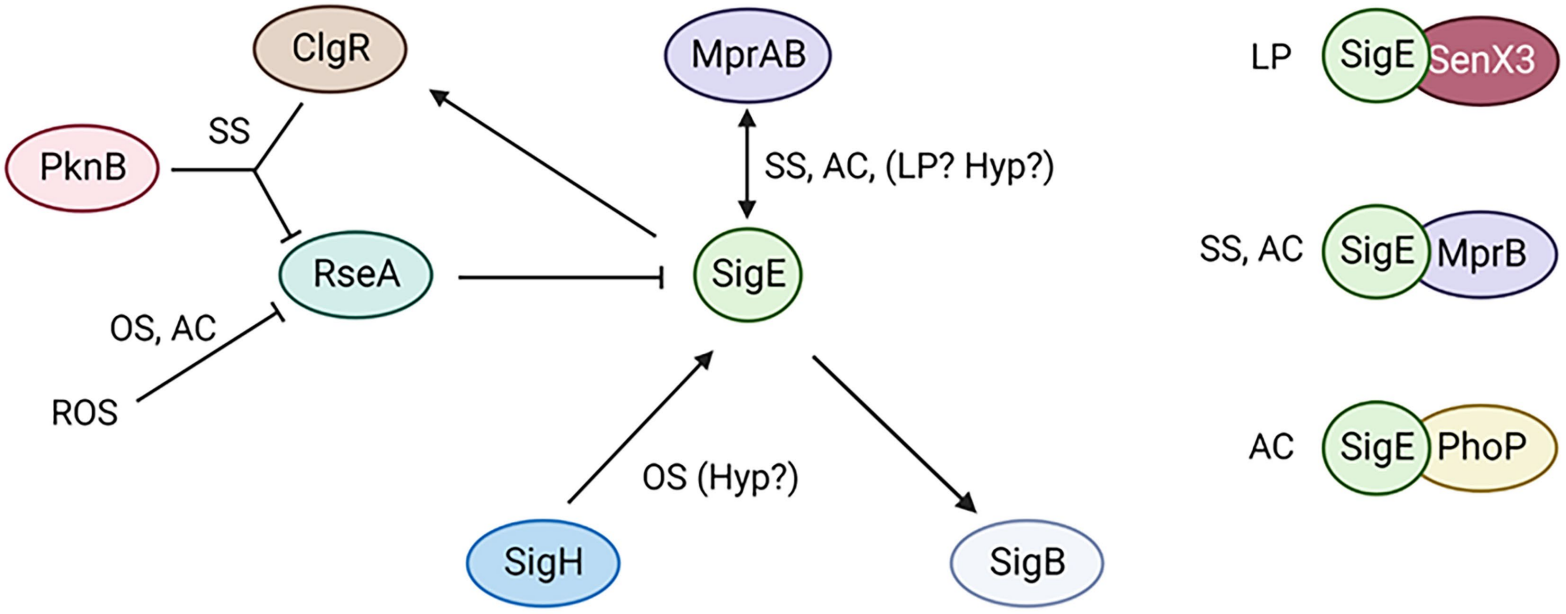
The SigE network is activated by different pathways depending on the conditions encountered by the bacteria. Moreover, SigE activity can be modulated at least by three different two component systems. SS, surface stress; AC, acidic pH; OS, oxidative stress; LP, low phosphate; Yyp, hypoxia. Created with BioRender.com.

SigE is one of the main master regulators of *M. tuberculosis*. The complex regulatory network governing SigE activity can be activated using different pathways in response to different environmental stimuli encountered by the bacteria during the infectious process. Moreover, once active SigE can interact with other regulators as the two-component systems MprAB, PhoP, or SenX3-RegX3 to activate different sets of genes depending on the environment encountered by the bacteria (Figure 5). Even if several aspects of SigE physiology are well known, there are still several aspects that need to be better investigated, as its involvement in stringent response, hypoxia and persistence. Understanding the architecture and the physiology of the SigE network can help to shed light on fundamental aspects of the host–parasite interaction strategy at the basis of the success of this formidable pathogen as (i) intracellular survival, (ii) survival in harsh environments as that encountered in caseum, (iii) the development of persisters able to escape drug treatment or (iv) the survival for decades in human tissues. The better understanding of these processes will provide new knowledge to develop new strategies to fight this old enemy.

## Author contributions

RM and RP conceived and contributed to write the manuscript. LC-M, GS, FB, and DS contributed to write the manuscript. MC and AS reviewed and approved the final version of the manuscript. All authors contributed to the article and approved the submitted version.

## Funding

RM laboratory is founded from the Innovative Medicines Initiative 2 Joint Undertaking (JU) under grant agreement no 853989, from Ministero dell’Università e della Ricerca Scientifica, Programmi di Ricerca Scientifica di Interesse Nazionale (PRIN) under grant agreement 20205B2HZE, and EU funding within the MUR PNRR Extended Partnership initiative on Emerging Infectious Diseases (project no. PE00000007, INF-ACT).

## Conflict of interest

The authors declare that the research was conducted in the absence of any commercial or financial relationships that could be construed as a potential conflict of interest.

## Publisher’s note

All claims expressed in this article are solely those of the authors and do not necessarily represent those of their affiliated organizations, or those of the publisher, the editors and the reviewers. Any product that may be evaluated in this article, or claim that may be made by its manufacturer, is not guaranteed or endorsed by the publisher.

## Footnotes

^1^Cioetto-Mazzabò, L., Babic, F., Boldrin, F., Sorze, D., Segafreddo, G., Provvedi, R., et al. Structure of the SigE regulatory network in Mycobacterium tuberculosis.
